# Early outpatient clinical experience with xanomeline and trospium chloride for schizophrenia: a case report

**DOI:** 10.3389/fpsyt.2025.1630574

**Published:** 2025-06-20

**Authors:** Maxwell Zachary Price, Richard Louis Price

**Affiliations:** ^1^ Department of Psychiatry, Hackensack Meridian School of Medicine, Nutley, NJ, United States; ^2^ Department of Psychiatry, Yale School of Medicine, New Haven, CT, United States

**Keywords:** xanomeline, trospium chloride, schizophrenia, outpatient, case series

## Abstract

The combination of xanomeline, a central/peripheral muscarinic agonist, and trospium chloride, a peripheral muscarinic antagonist, (X/T) was Food and Drug Administration (FDA) approved in September 2024 for schizophrenia in adults. FDA trial subjects experiencing exacerbation or relapse of psychotic symptoms, who were neither treatment-resistant nor had taken clozapine, were tapered off previous antipsychotics, or were treatment-naive, prior to rapid X/T titration in the hospital as monotherapy. This case series addresses real-world clinical questions about how to use X/T when comorbidities with schizophrenia are the rule, polypharmacy is commonplace, and discontinuing antipsychotics prior to X/T initiation is often infeasible due to safety concerns in outpatient settings. Based on our early experience treating 40 adult outpatients with schizophrenia and comorbidities using X/T to date, we present three representative cases to share the clinical pearls we have uncovered by applying extant preclinical and clinical data. To maximize efficacy while ensuring tolerability, it is important to track cholinergic (e.g., nausea, vomiting, and diarrhea) vs. anticholinergic (e.g., gastroesophageal reflux, constipation, and urinary retention) side effects, and to adjust X/T dose, titration, administration, and concomitant medications accordingly. X/T holds potential to improve cognitive deficits in comorbid autism or dementia, warranting further study.

## Introduction

Xanomeline and trospium chloride (X/T) was Food and Drug Administration (FDA) approved in September 2024 under the brand name Cobenfy™ (Bristol-Myers Squibb) (1), with three out of three positive trials in adults with schizophrenia experiencing an acute exacerbation or relapse of psychotic symptoms, who were neither treatment-resistant nor had taken clozapine. Trial subjects were tapered off and washed out from their prior antipsychotic treatment (89%) or were treatment-naive (11%) prior to a relatively rapid, 2-day titration from 50 mg xanomeline/20 mg trospium to 100 mg/20 mg, followed by another potential 5-day titration to 125 mg/30 mg in the hospital setting as a monotherapy (2–4).

Xanomeline is a central and peripheral, pseudo-irreversible, orthosteric (M1, M4), and allosteric (M4) cholinergic functional agonist at M1 and M4 receptors in the frontal cortex, hippocampus, basal ganglia, striatum, and laterodorsal tegmental nucleus (M4), and a partial agonist at M5 receptors in the midbrain. Activation of postsynaptic M1 receptors on GABA interneurons decreases neurotoxic glutamate, decreasing presynaptic dopamine release in the ventral tegmental area and potentially improving neuroplasticity, neurogenesis, neuroinflammation, learning, cognition, negative symptoms, and psychosis. Activation of M4 autoreceptors decreases acetylcholine, which decreases presynaptic dopamine release in the nucleus accumbens and ventral striatum, potentially improving psychosis, dyskinesia, and habit formation. Partial agonism at postsynaptic M5 receptors modulates dopamine in the forebrain, potentially improving negative symptoms, mood, and addictive behaviors (5, 6).

Notwithstanding the inpatient clinical trial data, for outpatient practitioners, the generalizability of the studied population and the treatment protocol is limited, leaving many real-world clinical questions about how to use X/T when comorbidities with schizophrenia are the rule, polypharmacy is commonplace, and discontinuing antipsychotics prior to X/T initiation is often infeasible due to safety concerns, such as psychotic relapse with potential risks to self or to others, in less-supervised community settings. Based upon our early experience treating 40 adult outpatients with schizophrenia and comorbidities using X/T to date, in our Hudson Valley, New York practice, we present three representative cases that are reflective of the entire cohort to illustrate the valuable clinical pearls we have uncovered through extrapolating and applying extant preclinical and clinical data. All patients were diagnosed as per DSM-5-TR diagnostic criteria based on clinical interviews with the patients and their caregivers. These three cases were selected because they reflect the nuances of managing schizophrenia with comorbidities and polypharmacy, a population that we commonly encounter in general outpatient practice but was not formally studied in the X/T FDA registration trials.

## Case presentation

Case 1 is an 18-year-old man who had struggled with social reciprocity, had poor eye contact, flat affect, preoccupation with idiosyncratic trivia (autism spectrum disorder), and eye blinking tics since youth. He subsequently developed trouble concentrating, auditory hallucinations with paranoia, and agitation for the previous 6 months, prompting him to drop out of his college engineering program. He first tried risperidone, which reduced his hallucinations and paranoia, but it was discontinued due to weight gain, gynecomastia, and dystonia. He then switched to aripiprazole, which was more effective for his negative symptoms, but it induced akathisia that did not respond to propranolol yet was resolved with benztropine. Nevertheless, he still had difficulty concentrating, which precluded him from returning to college, despite no prior history of attention deficit hyperactivity disorder. X/T was initiated at 50 mg/20 mg BID and benztropine was discontinued to avoid excessive anticholinergic effects in combination with trospium. While off benztropine, he continued to be free of akathisia despite continued aripiprazole dosing. However, he experienced violent nausea, vomiting, and diarrhea when he forgot to wait 2 hours after eating his late-night snack prior to his bedtime X/T dose (due to xanomeline’s peripheral cholinergic effects being unopposed, since trospium is not well absorbed with food) (7). Henceforth, we advise patients to take X/T 1 hour before breakfast and 1 hour before dinner to ensure proper dietary adherence as it is easier to remember when one started eating than when one finished. Ondansetron, a non-anticholinergic antiemetic, was later prescribed in conjunction with X/T to reduce GI upset, which began to subside after 2 weeks. Thereafter, he increased X/T to 100 mg/20 mg BID with return of the akathisia (xanomeline is an intestinal CYP3A4 inhibitor that can transiently raise aripiprazole levels) (7), so the aripiprazole dose was lowered with resolution of the akathisia, without psychotic relapse. Ostensibly counterintuitive, we have observed that pushing the X/T dose to the final maximum recommended dose of 125 mg/30 mg BID, once the previous dose is acceptably tolerated, can further improve the cholinergic gastrointestinal side effects of xanomeline due to the relatively greater proportional increase in the trospium component at this dose. After 4 weeks on 125 mg/30 mg, aripiprazole was discontinued. He is now back in college on X/T monotherapy with good concentration, psychosis free, tics resolved, solid eye contact, and improved social-emotional communication and insight, even exceeding his premorbid schizophrenia baseline functioning. He apologized for the suffering his illness had caused his parents, who graciously expressed, “We have our son back, and he’s better than ever!”

Case 2 is a 45-year-old married woman with chronic paranoid schizophrenia and worsening short and long-term memory, who failed multiple typical and atypical antipsychotics due to lack of efficacy and/or side effects. Over the years, she developed tardive dyskinesia and morbid obesity, for which she was taking a glucagon-like peptide (GLP-1) agonist with associated gastroparesis. She had been recently discharged from the hospital on olanzapine for an acute psychotic exacerbation with good effect but reported increased appetite, tremor, and exacerbated forgetfulness. She started X/T 50 mg/20 mg BID without food as per the package insert (7) but complained of constipation, gastroesophageal reflux (GERD), and urinary retention (potentially due to the excessive anticholinergic effects of trospium in combination with the anticholinergic effects from olanzapine). Taking X/T *with* food (to limit trospium absorption), along with 2 weeks of famotidine for the GERD, mitigated these side effects. After 100 mg/20 mg BID for 2 weeks, olanzapine was gradually tapered over 4 weeks with continued remission of psychosis and improved memory and executive functioning, with the tremor and gastroparesis resolved. She is back to playing piano after a 20-year hiatus due to her illness, and losing weight while off olanzapine and the GLP-1 agonist. Her husband appreciates being able to travel with her again, now that she has recovered her youthful *joie de vivre* from long ago.

Case 3 is a 75-year-old single woman with chronic paranoid schizophrenia with thought disorganization, Alzheimer’s dementia with agitation, and obsessive-compulsive disorder (OCD) with hand washing. She is a chronic cigarette smoker who failed lengthy state hospital stays, multiple antipsychotics, donepezil plus memantine, and electroconvulsive therapy (ECT), and currently takes clozapine and fluoxetine QHS with inadequate control of positive and negative symptoms, and residual OCD. Her family heard about the promise of X/T in the media before it was released and, in eager anticipation, she obtained samples the very first day it became available. She initiated X/T 50 mg/20 mg BID with food and developed nausea, which was alleviated by a dose reduction to 50 mg/20 mg QAM dosing (CYP2D6 inhibitors such as fluoxetine can raise xanomeline levels and duration, while trospium does not undergo CYP metabolism and is renally excreted) (7). After 2 weeks, X/T was raised to 100 mg/20 mg QAM and then, after another 2 weeks, clozapine was gradually tapered off over 6 weeks. She is no longer paranoid or agitated, her memory and communication are improved, her OCD has resolved, and she no longer feels cravings to smoke. Instead of sitting alone, mumbling to herself in a corner at the Adult Day Care Program, she now sings, dances, and engages with staff and peers.

## Discussion

In contrast to a 10:1 brain:plasma concentration for lipophilic xanomeline (5), trospium is a highly polar quaternary amine that has limited blood-brain barrier penetration and functions as an M1-M5 pan-muscarinic antagonist to mitigate the cholinergic side effects of xanomeline in the periphery when taken without food (8). When taken with food (off label), trospium is not well absorbed, leaving the peripherally cholinergic xanomeline unopposed, which we have found may be desirable under certain conditions, as referenced above, such as during cross-titrations from highly anticholinergic olanzapine, clozapine, or quetiapine. Eventually, tapering off these medications once X/T is at the therapeutic dose of 100 mg/20 mg BID (the maximum dose for seniors or with CYP2D6 inhibitors) (7) or 125 mg/30 mg BID is preferable as we have observed that their central anticholinergic effects can attenuate the central cholinergic benefits of X/T from being realized until these agents are discontinued (**Table 1**).

**Table 1:** Summary of clinical pearls.

	Side effects	Mitigation strategies
**Xanomeline**	Nausea/vomiting/diarrhea	1. No food 1 hour before X/T and at least 2 hours after finishing a meal 2. Slower X/T titration 3. Ondansetron BID prn 4. If on X/T 100 mg/20 mg, increase to 125 mg/30 mg
	With 2D6 inhibitors	1. Lower X/T dose BID 2. Change to QD dosing
	With 3A4 substrates	Lower dose of 3A4 substrate
**Trospium chloride**	Gastroesophageal reflux	Famotidine
	Constipation/urinary retention	Take with food
	With central anticholinergics (clozapine, olanzapine, and quetiapine)	Take with food, titrate X/T, then taper off anticholinergic
	With peripheral anticholinergics (diphenhydramine, benztropine, and oxybutynin)	Discontinue peripheral anticholinergics

X/T, xanomeline trospium chloride; BID, twice daily; QD, once daily.

Notwithstanding the parsimony of X/T monotherapy, we have also recognized that combining X/T (off label) with partial dopamine agonists, lurasidone, or lumateperone for associated mood symptoms, or with aripiprazole, risperidone, or paliperidone long-acting injectables (LAI) for adherence, is well tolerated as these classes of medications are not particularly anticholinergic. A recent *post hoc* analysis (9) of a 6-week outpatient study of adjunctive X/T for schizophrenia (NCT05145413) demonstrated no new safety signals and clinical improvement in combination with paliperidone (oral or LAI), aripiprazole (oral or LAI), ziprasidone, lurasidone, and cariprazine (nominal p-value=0.03, not adjusted for multiplicity) in contrast to risperidone (oral or LAI) (p-value=0.66). We found that medications such as risperidone and haloperidol, which can exacerbate negative symptoms, may mask the benefits of X/T until these medications are tapered off.

## Conclusion

We hope that sharing the particulars of these cases may help our fellow outpatient clinicians navigate the nuances of initiating and titrating X/T to maximize efficacy while ensuring tolerability. As the first FDA-approved medication for schizophrenia psychosis not classified as an “antipsychotic”, X/T does not carry the familiar boxed warning related to increased mortality in elderly patients with dementia-related psychosis. Furthermore, unlike all antipsychotics for schizophrenia that either completely or partially block dopamine receptors, X/T modulates presynaptic dopamine through muscarinic receptors, with low risk for weight gain, sedation, prolactin elevation, QT prolongation, cardiometabolic or extrapyramidal side effects, and significant improvements noted in positive, negative, cognitive, and general psychopathology symptoms in clinical trials (10). We recommend keeping careful track of transient cholinergic (e.g., nausea, vomiting, and diarrhea) vs. anticholinergic side effects (e.g., constipation and urinary retention), and adjusting the X/T dose, titration, administration, and concomitant medications accordingly. This personalized approach can help our patients not just settle for “stable” symptoms, but reach for a higher level of “optimal” functioning—the clarity of mind that so often eludes individuals suffering from schizophrenia along with common comorbidities, such as autism spectrum disorder and dementia.

The limitations of this study include the observational nature of the data, the potential for selection bias in choosing representative cases, and the lack of psychometric data at baseline and at final estimation. While the results of this case series are promising, more studies with larger numbers of participants are needed to confirm these findings.

## Data Availability

The original contributions presented in the study are included in the article/Supplementary Material. Further inquiries can be directed to the corresponding author.
